# Genetic Characteristics of Methicillin-Resistant Staphylococcus argenteus Isolates Collected in the Dutch National MRSA Surveillance from 2008 to 2021

**DOI:** 10.1128/spectrum.01035-22

**Published:** 2022-08-25

**Authors:** Sandra Witteveen, Antoni P. A. Hendrickx, Angela de Haan, Daan W. Notermans, Fabian Landman, Marga G. van Santen-Verheuvel, Sabine C. de Greeff, Ed J. Kuijper, Noortje M. van Maarseveen, Saara Vainio, Leo M. Schouls

**Affiliations:** a Centre for Infectious Disease Control (CIb), National Institute for Public Health and the Environment (RIVM), Bilthoven, the Netherlands; b Department of Medical Microbiology and Medical Immunology, Rijnstate Hospital, Velp, the Netherlands; c Department of Medical Microbiology, Immunology and Infection Control, St Antonius Hospital, Nieuwegein, the Netherlands; Quest Diagnostics

**Keywords:** MRSArg, SCC*mec* type IV, SCC*mec* subtype IVc(2B), resistance genes, enterotoxins, *Staphylococcus argenteus*, virulence factors

## Abstract

Staphylococcus argenteus is a recently described member of the Staphylococcus aureus complex (SAC) and is associated with human disease. The frequency and intensity of infections caused by *S. argenteus* are similar to those of Staphylococcus aureus. *S. argenteus* can harbor antibiotic resistance genes and a variety of virulence factors analogous to methicillin-resistant S. aureus (MRSA). The aim of our study was to analyze a collection of isolates in the Dutch national MRSA surveillance from January 2008 until March 2021 that were nontypeable by multilocus variable-number tandem-repeat analysis (MLVA). Matrix-assisted laser desorption ionization–time of flight mass spectrometry (MALDI-ToF MS) was used for identifying the *S. argenteus* isolates, and whole-genome sequencing and SeqSphere were used to generate an in-house whole-genome multilocus sequence typing (wgMLST) scheme for typing the isolates. Furthermore, the presence of antibiotic resistance genes, replicons, and virulence genes was determined. Of 52,467 isolates submitted as MRSA from January 2008 until March 2021, 64 isolates (0.12%) were nontypeable with MLVA, and 54 of them were identified with mass spectrometry (MALDI-ToF MS) as *S. argenteus*. It appeared in retrospect that the first methicillin-resistant *S. argenteus* (MRSArg) was already submitted in 2008. An in-house-developed *S. argenteus* wgMLST scheme revealed that *S. argenteus* isolates clustered in 5 genomic groups which were characterized by distinct MLST types, resistomes, plasmid replicon families, and virulence factors. All but one isolate carried the staphylococcal chromosomal cassette *mec* (SCC*mec*) type IV harboring the methicillin resistance gene *mecA* and represent MRSArg. Most of the isolates with SCC*mec* subtype IVc(2B) had a trimethoprim resistance gene, *dfrG*, and harbored a *blaZ*-carrying plasmid, and most MRSArg isolates have the immune-modulating genes *scn* and *sak*. Nine of the 47 isolates carried enterotoxin-encoding genes *seg*, *sei*, *sem, seo*, and *seu*, which might be able to cause food poisoning. In some persons there was long-term persistence of MRSArg, and there were several genetically related MRSArg isolates in people living in close proximity, suggesting direct human-human transmission.

**IMPORTANCE** We show that MRSArg has been circulating in the Netherlands since at least 2008. Although MRSArg is distinct from MRSA, it has a comparable population structure and carries similar resistance and virulence genes. The Dutch national MRSA surveillance has been expanded to include other methicillin-resistant members of the S. aureus complex, such as *S. argenteus* and Staphylococcus schweitzeri.

## INTRODUCTION

Staphylococcus argenteus belongs to the Staphylococcus aureus complex (SAC) together with Staphylococcus schweitzeri, S. aureus, and the recently described Staphylococcus roterodami (1) and Staphylococcus singaporensis (2). *S. argenteus* was first described in 2006 (3) and was shown to belong to multilocus sequence typing (MLST) clonal complex 75 (CC75) of S. aureus. However, a later study showed that these CC75 isolates were distinct from S. aureus in several ways, and therefore, they were designated a separate species in 2015 (4), namely, *S argenteus*. CC75 comprises several MLST types exclusively found in *S. argenteus* isolates, e.g., ST2250, ST1223, ST2198, and ST2854 (4–7). In 2011 it was found that isolates with other sequence types (STs) not belonging to CC75, e.g., ST2196, ST1594, and ST2793, were genetically very similar to *S. argenteus* when their genomes were compared (5). Nowadays, *S. argenteus* isolates belonging to more than 60 different STs, from five different clonal complexes, have been identified (6, 8). *S. argenteus* has been found in many countries all over the world, including in the Netherlands (3–20).

Initially it was thought that *S. argenteus* was less virulent than S. aureus, because it lacks staphyloxanthin (5, 14). However, a study in 2015 (9) demonstrated that *S. argenteus* can cause health issues and that the frequency of infections caused by *S. argenteus* is similar to that of S. aureus*. S. argenteus* can cause skin, bone, and joint infections (3, 6, 10), bloodstream infections (11), and toxin-mediated syndromes, like staphylococcal food poisoning (12). Other studies (7, 13–15) have shown that *S. argenteus* can be resistant to various classes of antibiotics due to the presence of antibiotic resistance genes, e.g., *blaZ*, *fusA*, *tet(L)*, or *aph(3*′*-III)* (7, 8, 13, 15, 20), and that there are methicillin-susceptible *S. argenteus* (MSSArg) but also methicillin-resistant *S. argenteus* (MRSArg) isolates harboring SCC*mec* type IV containing *mecA* (8). A variety of virulence factors have been found in *S. argenteus*, including the Panton-Valentine leukocidin (PVL) gene and the five classical enterotoxins (6, 8, 12, 14–16, 20), which may have a clinical relevance.

In a previous study, we showed that a number of isolates that were submitted for the Dutch national methicillin-resistant S. aureus (MRSA) surveillance were MRSArg and that MRSArg had been detected in the Netherlands as early as 2008 (17).

From January 2008 until March 2021, 52,467 MRSA isolates were submitted for the Dutch national methicillin-resistant S. aureus (MRSA) surveillance that were subjected to multilocus variable-number tandem-repeat analysis (MLVA). However, 54 isolates from 47 different persons were determined as nontypeable for MLVA and were shown to be MRSArg.

In the study presented here, we performed next-generation sequencing (NGS) and third-generation sequencing (TGS) to assess the genetic relationships and to determine molecular characteristics of these Dutch MRSArg isolates.

## RESULTS

### Identification and geographic distribution of *S. argenteus* in the Netherlands.

Between January 2008 and March 2021, the National Institute for Public Health and the Environment (RIVM) received 52,467 presumed MRSA isolates of which 64 were nontypeable by MLVA (0.12%). All 64 isolates were analyzed by next-generation sequencing (NGS) and matrix-assisted laser desorption ionization–time of flight mass spectrometry (MALDI-ToF MS). In the mass spectrometry (MALDI-ToF MS), 54 isolates of the 64 nontypeable isolates (84%) appeared to belong to the Staphylococcus genus with the highest score being for *S. argenteus*, six isolates were classified as S. aureus, and four isolates were other species (Staphylococcus sciuri and three Gram-negative isolates). These results were corroborated by analysis of the NGS data. The six previously nontypeable S. aureus isolates yielded complete MLVA profiles in the MLVA of freshly made bacterial lysates. The 54 *S. argenteus* isolates were obtained from 47 different persons; from five persons two isolates were obtained and from one person three isolates were obtained (Table 1). For this study 47 unique isolates were used, the first isolate of each person.

**TABLE 1:** Metadata of the Dutch *S. argenteus* isolates used in this study

Isolate	*S. argenteus* PCR	Person^*a*^	Age (yr)	Yr of sampling	Submitter of sample	Specimen^*b*^	Carrier or infection	Risk detail and/or reason for culturing
RIVM_M003546	+	Person 1	25	2008		Swab (T)	Carrier	
RIVM_M011864	+	Person 1	28	2010		Swab (N, P, T)	Carrier	
RIVM_M025118	+	Person 2	39	2014		Other human material	Unknown	
RIVM_M026823	+	Person 2	40	2014		Urine	Infection	
RIVM_M031425	+	Person 3	9	2015	General practice	Pus	Infection	
RIVM_M031426	+	Person 3	9	2015	General practice	Pus	Infection	
RIVM_M040661	+	Person 4	18	2017	General practice	Swab (T)	Carrier	None
RIVM_M040662	+	Person 4	18	2017	General practice	Swab (N)	Carrier	None
RIVM_M040163	+	Person 5	31	2017	Hospital	Swab (N)	Carrier	Screening, was nursed in Australia
RIVM_M041166	+	Person 5	31	2017	Rehabilitation	Swab (N)	Carrier	Screening, known MRSA carrier
RIVM_M045471	+	Person 5	32	2018	General practice	Swab (T)	Carrier	
RIVM_M046211	+	Person 6	28	2019	General practice	Swab (N)	Carrier	
RIVM_M046343	+	Person 6	28	2019	Hospital	Swab (P)	Carrier	
RIVM_M002983	+		60	2008		Tumor thump	Unknown	
RIVM_M008822	+		61	2010		Swab (N, P, T)	Carrier	
RIVM_M019208	+		NA^*d*^	2012		Swab of ICD^*e*^ pocket	Unknown	
RIVM_M020832	+		36	2013		Swab (T)	Carrier	
RIVM_M023607	+		33	2013		Wound fluid	Unknown	
RIVM_M024573	+		27	2014		Wound fluid	Infection	
RIVM_M025002	+		49	2014		Pus	Infection	
RIVM_M025606	+		29	2014		Other human material	Unknown	
RIVM_M025618	+		28	2014		Swab (N, P, T)	Carrier	
RIVM_M027397	+		56	2014		Swab (N)	Carrier	
RIVM_M029687	+		70	2015	Hospital	Swab (P, T)	Carrier	Screening, person was nursed in Belgium^*c*^
RIVM_M029794	+		50	2015		Sputum	Infection	
RIVM_M030565	+		42	2015		Swab (N)	Carrier	
RIVM_M030644	+		20	2015	Hospital	Wound fluid	Infection	Screening, vacation in Philippines
RIVM_M035744	+		69	2016	General practice	Groin swab	Unknown	
RIVM_M036020	+		25	2016		Swab (N)	Carrier	
RIVM_M036044	+		62	2016	Hospital	Wound fluid	Infection	
RIVM_M036477	+		40	2016	Hospital	Wound fluid	Infection	Coincidental finding in patient/client
RIVM_M036821	+		33	2016	Hospital	Drain fluid	Unknown	Coincidental finding in patient/client
RIVM_M037020	+		34	2016	Hospital	Swab (P, T)	Carrier	Screening, had had contact with MRSA^+^ person
RIVM_M037112	+		44	2016	Hospital	Wound	Unknown	
RIVM_M038289	+		48	2017		Swab (T)	Carrier	
RIVM_M038319	+		46	2017	Hospital	Wound fluid	Infection	Coincidental finding in patient/client
RIVM_M040187	+		19	2017	Hospital	Swab (N)	Carrier	
RIVM_M041614	+		37	2017	General practice	Pus	Infection	
RIVM_M042032	+		51	2018	General practice	Swab (P)	Carrier	Screening, was nursed in Philippines^*c*^
RIVM_M042169	+		40	2018	Practice/clinic/treatment center	Swab (T)	Carrier	
RIVM_M042294	+		22	2018	Hospital	Swab (N)	Carrier	
RIVM_M042580	+		10	2018	General practice	Swab (T)	Carrier	Screening, from Somalia, here for family reunion
RIVM_M042731	+		29	2018	General practice	Swab (R)	Carrier	Screening, had had contact with MRSA^+^ person
RIVM_M043825	+		43	2018	Practice/clinic/treatment center	Swab (T)	Carrier	
RIVM_M044970	+		65	2018	Hospital	Swab (N)	Carrier	
RIVM_M046950	+		25	2019	Hospital	Swab (T)	Carrier	
RIVM_M046968	+		66	2019	Hospital	Blood	Infection	
RIVM_M081107				2018				
RIVM_M083837	Not tested		37	2020	Hospital	Swab (N, P, T)	Carrier	
RIVM_M085152	Not tested			0	2020		Hospital swab (T)	Carrier	
RIVM_M085212	Not tested			39	2020	Health service	Swab (N, T)	Carrier	
RIVM_M087541	Not tested		64	2021	Hospital, intensive care	Sputum	Infection	
RIVM_M087650	Not tested		66	2021	Hospital, intensive care	Swab (P)	Carrier	Screening
RIVM_M088004	Not tested		64	2021	Hospital, nursing ward	Swab (T)	Carrier	Screening

a There were 6 persons with multiple isolates; these isolates are numbered person 1 to 6. All other isolates were obtained from different persons.

b The different swab locations used for sampling (N, nose; P, perineum; R, rectum; T, throat).

c The person was nursed in a health care facility in a foreign country, less than 2 months previously, for less than 24 h.

d NA; not applicable.

e ICD pocket, implantable cardioverter defibrillator pocket.

Twenty-three of the 47 isolates were obtained from persons living in the more densely populated provinces of the Netherlands, Utrecht and North and South Holland, 12 were from other provinces, three isolates were retrieved from the Dutch Caribbean island of St. Maarten, and for nine isolates this information was missing. The epidemiological questionnaires showed that the reason for sampling was screening for MRSA carriage based on risk factors (27/47; 57.5%) or clinical indication (11/47; 27.7%), and some isolates had an incomplete submission form (8/47; 17.0%). One isolate (1/47; 2.1%) was reported to be obtained from an implantable cardioverter defibrillator pocket. Most of the MRSA carriage screening samples were a swab from nose, perineum, rectum, or throat (29/47; 61.7%), and 34.0% (16/47) of the isolates came from other clinical materials such as blood, pus, sputum, urine, and wound fluid (Table 1).

### wgMLST revealed distinct genogroups in the *S. argenteus* minimum spanning tree (MST).

**FIG 1:**
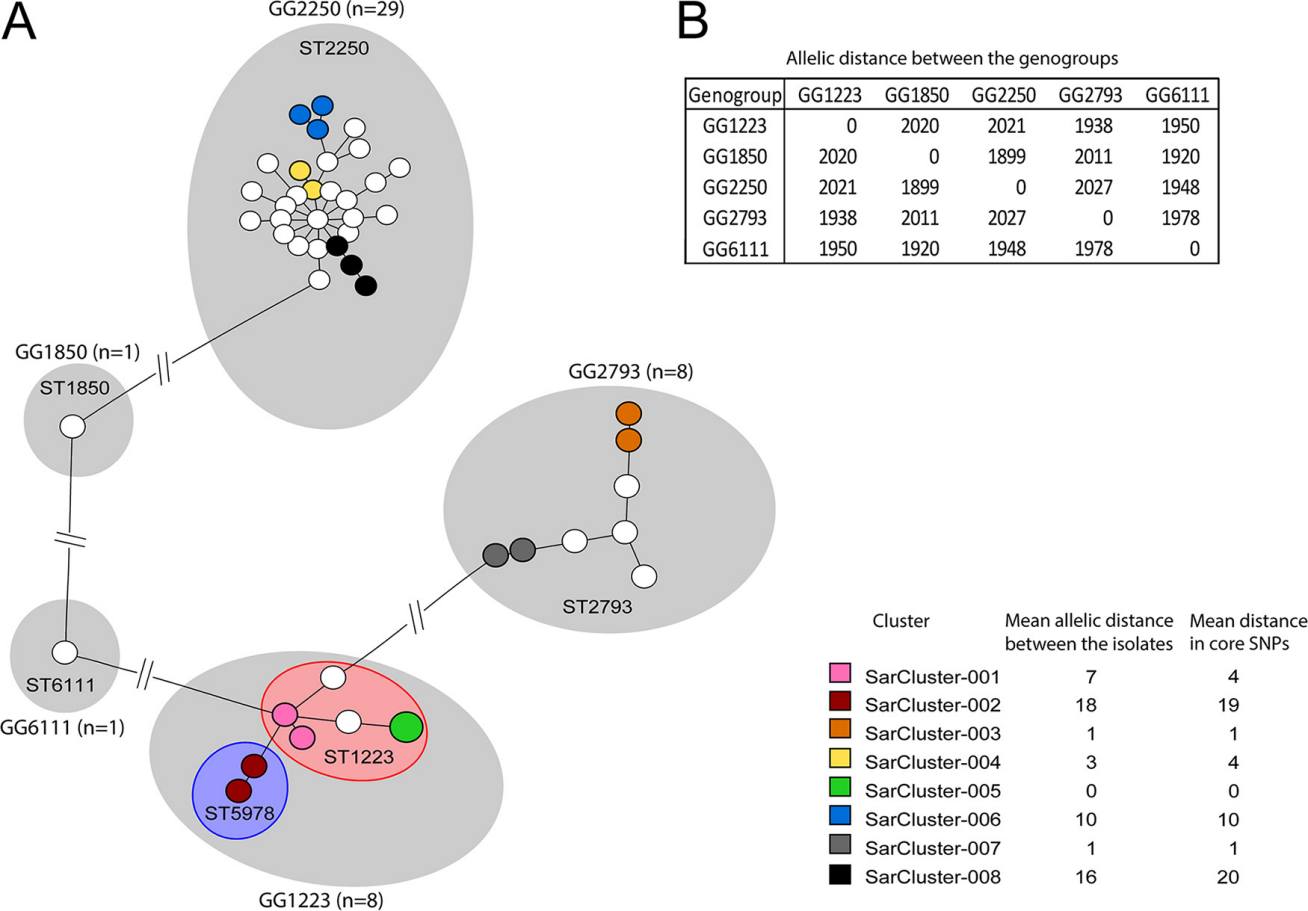
The wgMLST-based population structure of Dutch *S. argenteus* isolates. (A) MLST sequence types and wgMLST genogroups are indicated in the minimum spanning tree (MST). Each circle represents the first unique isolate per person, and the circle size indicates the number of isolates. The colors of the circles represent genetic clusters (right bottom). Genogroups (GG) are indicated with gray halos, while specific MLST STs are highlighted with colored halos. A genetic cluster of *S. argenteus* isolates is defined as ≥2 isolates that differ by ≤20 alleles. (B) Overview of the allelic distances between the genogroups from the wgMLST MST.

Whole-genome MLST (wgMLST) of the 47 Dutch *S. argenteus* isolates revealed that they were partitioned into five groups, designated as genogroups (GGs), with a pairwise difference between genogroups of more than 1,800 alleles (Fig. 1A and B) and with a pairwise distance of isolates within a genogroup of less than 145 alleles. The isolates within each genogroup had the same classical S. aureus MLST sequence type, except for genogroup GG1223, which comprised two sequence types, ST1223 and ST5978 (Fig. 1A). The isolates belonging to these two MLST types carried different alleles of the MLST gene *tpi* only, and this difference is based on a single mutation at position 186 in this gene, indicating that these two sequence types are very similar. One *S. argenteus* strain with a new allelic profile was found and was assigned MLST ST6111. The different *S. argenteus* GGs were characterized by distinct resistomes, plasmid replicon families, and virulence factors (Table 2).

**TABLE 2:**
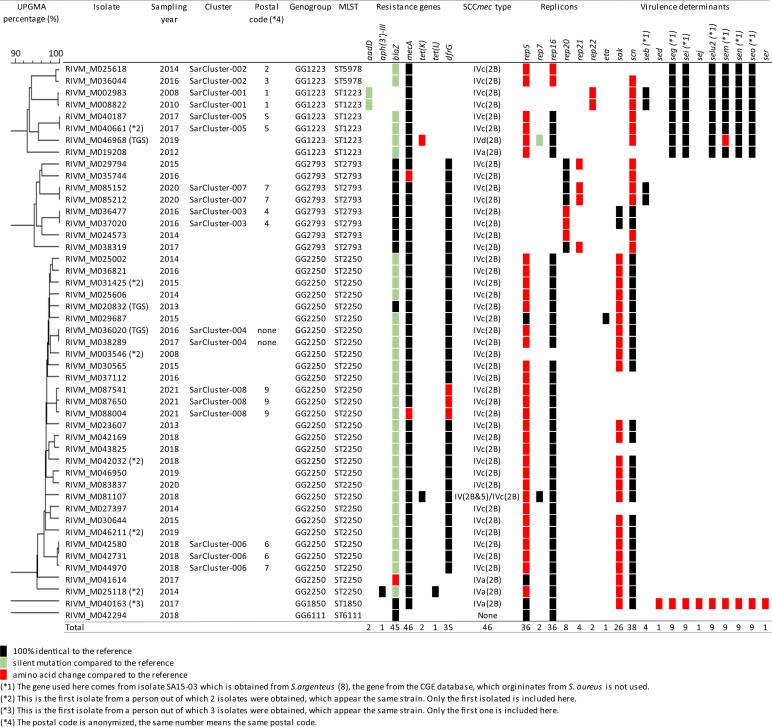
Genetic characteristics of the 47 unique Dutch *S. argenteus* isolates

The set of isolates was also analyzed using core single nucleotide polymorphism (SNP) typing (see Fig. S1 and Table S1 in the supplemental material). The distribution of genogroups and the genetic clusters are alike when comparing core SNPs with wgMLST. Only the genetic difference between the genogroups is much larger, more than 11,000 SNPs compared to an allelic difference of 1,800.

### Evidence of long-term carriage of *S. argenteus* strains.

For six persons multiple isolates were submitted over time, which enabled intraperson analysis of the genetic variation of *S. argenteus* strains. The multiple isolates were obtained with different time periods between samplings, ranging from 0 days in person 4 to 834 days in person 1 (Table 3). In five of the six persons from whom multiple isolates were obtained, the wgMLST profiles within the isolate sets differed in a maximum of three alleles (3/2,555; 0.12%) independent of the time between the person’s samplings (Table 3). In addition, the resistomes and replicons within each isolate set were identical. The wgMLST profile of two isolates obtained from person 1 differed in 19 alleles (19/2,555; 0.74%), suggesting this person had acquired a different MRSArg strain. This idea was supported by the fact that the first isolate did not carry the *blaZ* resistance gene and the rep5 and rep16 replicons but did carry the immune-modulating genes *sak* and *scn*. The second isolate had a *blaZ* resistance gene and the rep5 and rep16 replicons but did not carry the immune-modulating genes *sak* and *scn*. However, the *blaZ* gene is located on a rep5-rep16 plasmid and the *sak* and *scn* genes are located on the Sa3 phage. Therefore, the second isolate may also represent the same MSRArg strain that had acquired a *blaZ* plasmid and had lost the Sa3 phage.

**TABLE 3:**
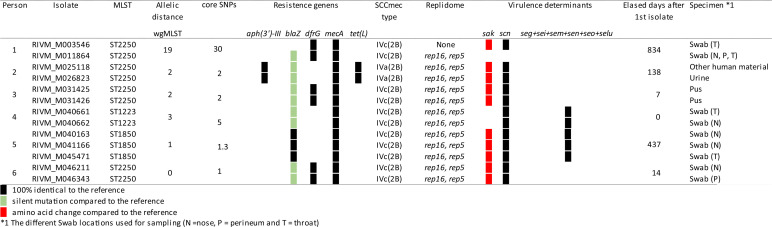
Genetic characteristics of duplicate Dutch *S. argenteus* isolates found in 6 persons

The multiple isolates from one person were also analyzed using core SNP typing (Table 3 and Table S1). The allelic differences found with wgMLST and the number of core SNPs are alike; only the two isolates from person 1 seemed to have a higher number of SNPs (30 SNPs) compared to the allelic differences found with wgMLST (19 wgMLST alleles).

### Transmission of *S. argenteus* in the Netherlands.

Eight groups comprising 18 *S. argenteus* isolates were highly related and designated genetic clusters SarCluster-001 to SarCluster-008 (Fig. 1A). Within a genetic cluster the mean allelic distance between two isolates was 18 or less, with a maximum pairwise distance between two isolates of 19 alleles. The persons from whom *S. argenteus* isolates were obtained belonged to the same genetic cluster and lived within the same 4-digit postal code region, except for the two isolates of SarCluster-002 and the third isolate of SarCluster-006 (Table 3). Isolates from SarCluster-007 were obtained from a father and his newborn child, and SarCluster-008 isolates were retrieved from three men who shared a household. Isolates from SarCluster-003 were obtained from persons from the small Caribbean island of St. Maarten, but detailed residential data were lacking. Except for one isolate in SarCluster-008, with one single nucleotide polymorphism (SNP) in *mecA*, the isolates belonging to the same genetic cluster had identical resistance genes, replicons, SCC*mec* types, and subtypes as well as the same virulence factors (Table 2), suggesting the genetic clusters represent interperson *S. argenteus* transmissions.

The isolates that form a genetic cluster were also analyzed using core SNP typing (Fig. 1, Fig. S1, and Table S1). The allelic differences found with wgMLST and the number of core SNPs are alike.

### Genomic comparison of *S. argenteus* isolates from the Netherlands with isolates from other countries.

**FIG 2:**
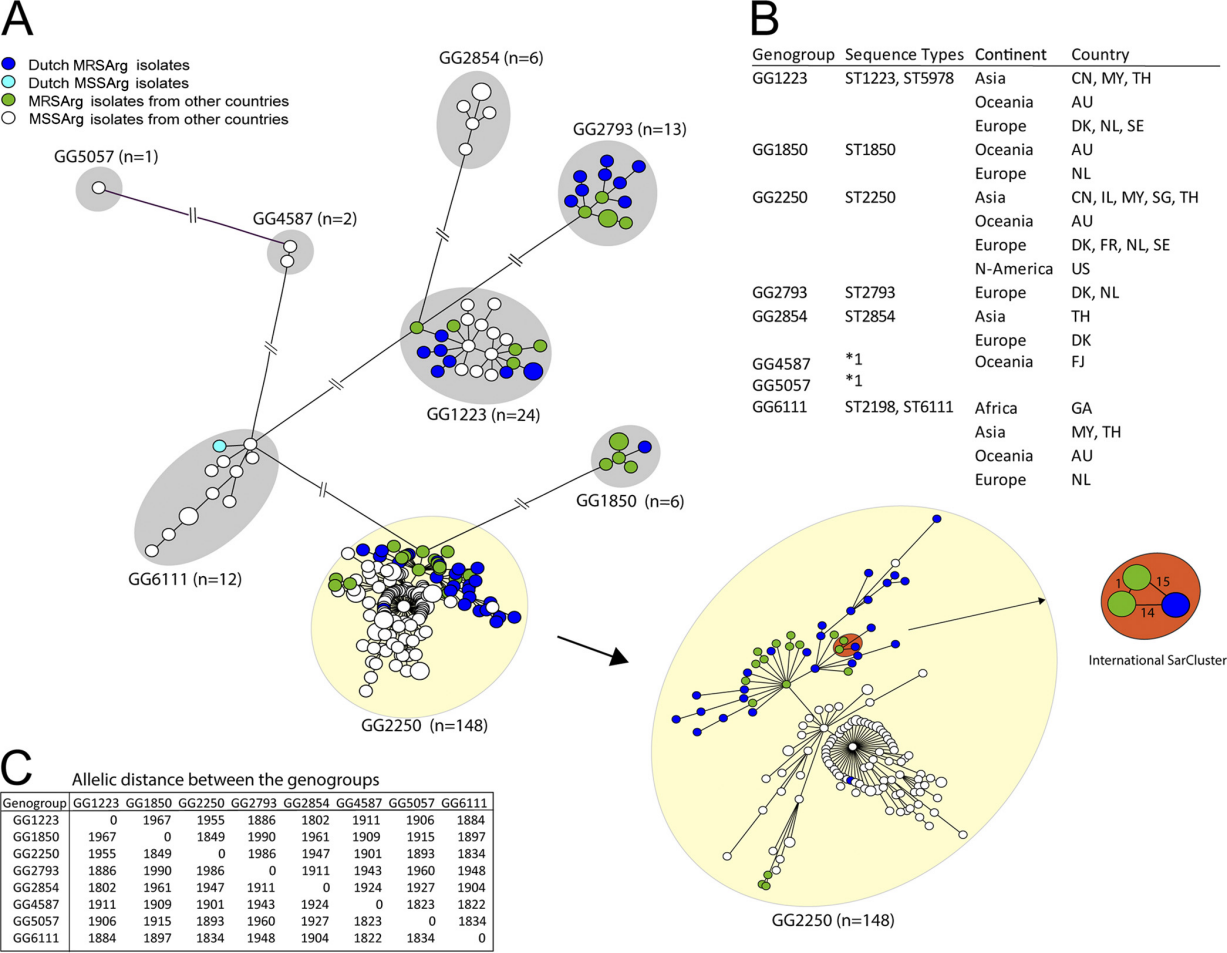
International comparison of *S. argenteus* isolates by wgMLST. (A) In-house wgMLST MST of *S. argenteus* obtained in the Netherlands and from other countries, visualized in a minimum spanning tree (MST) with only the first unique isolate per person. GG indicates genogroups, which are encircled with gray halos, except for GG2250, which is light yellow. The Dutch MRSArg isolates are colored dark blue, and the Dutch MSSArg isolates are colored light blue. The MRSArg isolates from other countries are green, while the MSSArg isolates from other countries are white. An international genetic cluster with an *S. argenteus* isolate from the Netherlands and two isolates from the United States is depicted in red. (Inset) Closeup of GG2250 to provide greater detail of the international *S. argenteus* cluster. (B) Overview of the genogroups and isolates from different countries in the genogroups. The ISO 3166 code is used for country abbreviation. *1, these isolates had an incomplete MLST profile. The sequence type nearest the incomplete profile is used as the genogroup name. (C) Overview of the allelic distances between the genogroups.

Comparison of the wgMLST profiles of the 47 *S. argenteus* isolates retrieved in the Netherlands with the wgMLST profiles of 167 isolates collected in other countries showed a highly similar distribution of genogroups (Fig. 2A). Eight genogroups were identified which differed by more than 1,800 alleles from each other (Fig. 2A and C), and isolates within a genogroup had a pairwise distance of less than 200 alleles. As with the Dutch *S. argenteus* isolates, most isolates from other countries belonged to GG2250 (Fig. 2A and B). A more detailed view of GG2250 showed two subgroups within the genogroup. Except for one isolate, all Dutch isolates resided in the upper branch of GG2250 with isolates from Denmark and Sweden and with two isolates sequenced in the United States. Most genogroups have a global composition, with isolates from several different continents, except for genogroup GG2793 (*n* = 13), which was comprised of European isolates only. Genogroups GG1850 and GG2854 carried isolates from two countries only. GG2793 and GG1850 contained only MRSArg isolates, whereas genogroups GG1223 and GG2250 have both MRSArg and MSSArg isolates. GG2250 was comprised of two subgroups, and the upper branch were mainly MRSArg isolates while the lower branch contained mainly MSSArg isolates. The other genogroups were comprised of only MSSArg isolates, and the only Dutch MSSArg isolate belongs to GG6111 together with other MSSArg isolates from other countries. In GG2250, a genetic cluster with a maximum allelic difference of 15 was identified with one Dutch isolate, RIVM_M025002, and two internationally obtained isolates (GenBank accession numbers JGHK00000000 and JGMK00000000, red circle in Fig. 2A). These isolates shared the same resistance genes, SCC*mec* type, and virulence factors. Isolate JGMK00000000 differed from the other two isolates because it harbored two replicons that were absent in the other isolates (data not shown). This was the only identified genetic cluster containing *S. argenteus* isolates from the Netherlands and other countries.

### The resistome of *S. argenteus*.

**FIG 3:**
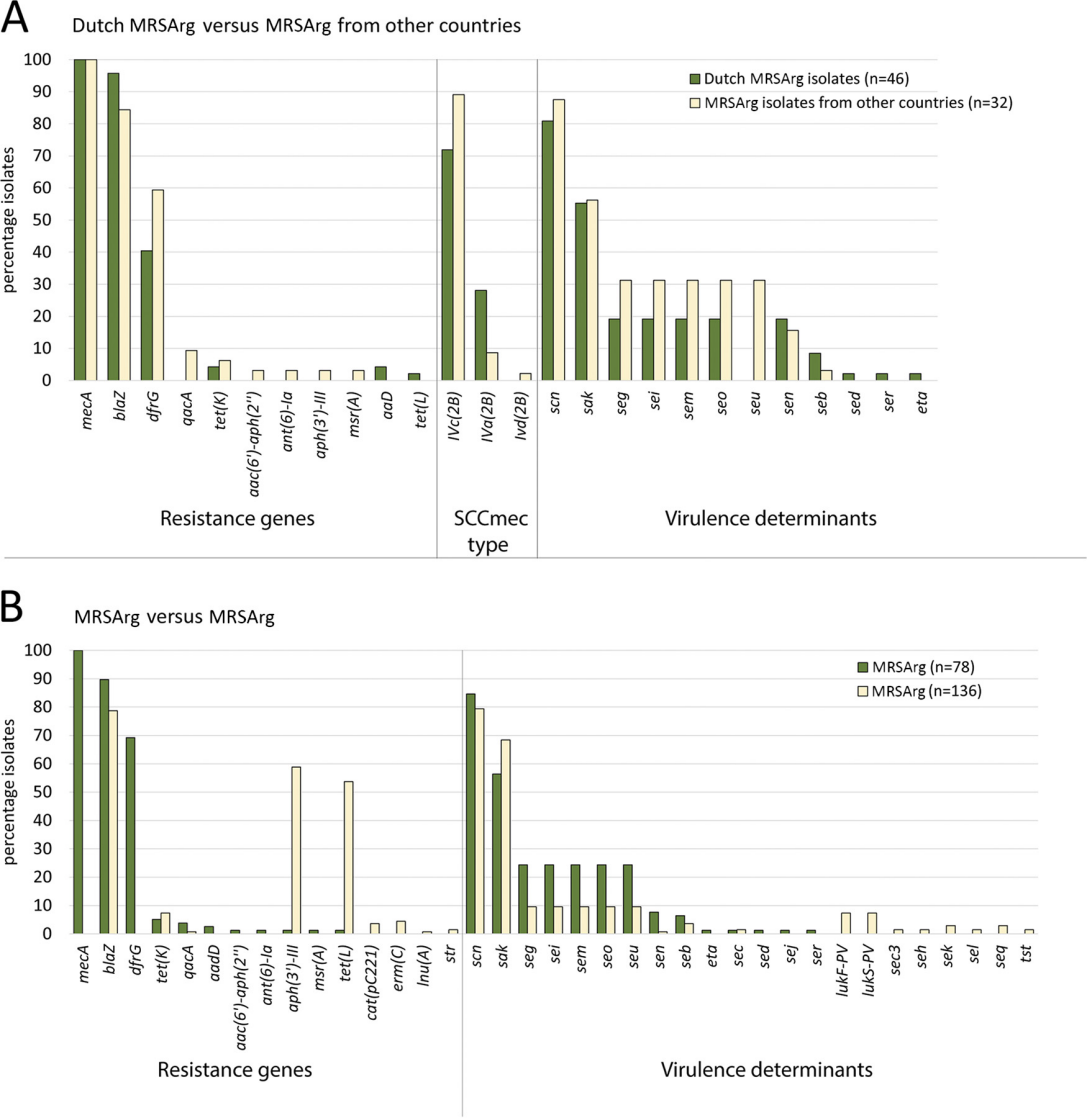
International comparison of SCC*mec* type, resistance genes, and virulence determinants of *S. argenteus*. (A) Resistance genes, virulence factors, and SCC*mec* cassette types as identified through ResFinder, VirulenceFinder, and SCCmecFinder found in the Dutch MRSArg isolates compared with the findings in MRSArg isolates from other countries. (B) Resistance genes and virulence factors as identified through ResFinder and VirulenceFinder found in all the MRSArg isolates compared with the findings in all MSSArg isolates.

Forty-six of the 47 *S. argenteus* isolates (98%) from the Netherlands harbored a *mecA* gene and had the SCC*mec* type IV cassette (Fig. 3A). Thirty-five of the 41 isolates (85%) with the SCC*mec* type IVc(2B) also had a trimethoprim resistance gene, *dfrG*, embedded into the SCC*mec*, and this gene was found only in isolates with SCC*mec* type IVc(2B). Of all isolates studied in other countries only 32 of the 167 isolates (19.2%) were MRSArg. When comparing the Dutch MRSArg isolates with the MRSArg isolates from other countries, we observed that more isolates from other countries had the *dfrG* gene and the SCC*mec* type IVc(2B) (Fig. 3A). Next, we observed resistance genes *aadD* and *tet(L)* in a few Dutch MRSArg isolates while they were absent in isolates from other countries. Vice versa, the *qacA*, *aac(6′)-aph(2′')*, *ant(6)-Ia*, *aph(3′)-III*, and *msr(A)* genes were observed only in isolates from other countries. The *tet(K)* gene was found in MRSArg isolates in the Netherlands as well as in MRSArg isolates from other countries. When comparing the MRSArg isolates with MSSArg isolates, most MSSArg isolates had *blaZ* (78.7%), comparable to the MRSArg isolates (89.7%) (Fig. 3B). Two resistance genes, *aph(3′)-III 3* and *tet(L)*, were observed more often in MSSArg isolates (59% and 54%) than in MRSArg isolates (1% and 1%), respectively.

### Virulence determinants of MRSArg and MSSArg.

When comparing the Dutch MRSArg isolates with the MRSArg isolates from other countries, we observed that 55% of the isolates had a *sak* gene (Fig. 3A). The *scn* gene was found in 80.6% of the Dutch MRSArg isolates and 87.5% of the MRSArg isolates from other countries. MRSArg isolates from other countries contained more putative enterotoxin-encoding genes *seg*, *sei*, *sem*, *seo*, and *seu* than did the Dutch MRSArg isolates. The percentage identity of the *seg*, *sei*, *sem*, *sen*, *seo*, and *seu* genes in the Dutch MRSArg isolates were less than 97%, suggesting these represent novel variants. The six putative enterotoxin genes found in the Dutch MRSArg isolates were analyzed by BLAST, revealing 100% similarity with enterotoxin genes found in the *S. argenteus* strain MSHR1132 (GenBank accession number FR821777) described by Wakabayashi et al. (12), but they used the name *selu2* instead of *seu*. It appears that the enterotoxin-encoding genes in the only Dutch MSSArg isolate are slightly different from those found in the MRSArg isolates. Furthermore, both MRSArg and MSSArg groups contained the *scn* gene in 80% of the isolates (Fig. 3B). The MSSArg isolates contained the *sak* gene (68.3%) more often than did MRSArg isolates (56.4%). MRSArg contained enterotoxin-encoding genes *seg*, *sei*, *sem*, *seo*, and *seu* (24.4%) more often than did MSSArg (9.6%). The PVL-encoding genes were identified in the MSSArg isolates (7.4%), but these were absent in MRSArg isolates.

## DISCUSSION

Here, we report the identification and genomic characterization of methicillin-resistant *S. argenteus* isolates that were misidentified and submitted as MRSA to the Dutch national MRSA surveillance in the period from January 2008 until March 2021.

MRSArg has been circulating in the Netherlands since at least 2008 and is different from MRSA; however, it has a comparable population structure and harbors similar resistance and virulence genes.

We cannot estimate the prevalence of MRSArg in the Netherlands, but nevertheless our study shows that MRSArg carriage and transmission do occur, which merits further study.

Using an in-house wgMLST scheme for *S. argenteus*, we found a division of the Dutch MRSArg isolates into five different genogroups, which differed in MLST sequence types, resistance genes, replicon families, and virulence determinants. A similar division was found in the *S. argenteus* isolates from other countries obtained from the NCBI and SRA databases, suggesting global spread of *S. argenteus* strains. Previously the identification of six clusters of *S. argenteus* was described with phylogenetic analysis (14), and later seven *S. argenteus* clades were described with the use of SNP typing with 52,329 SNPs (6). The identified genogroups in this study have a distribution similar to those in the work of Kaden et al. (14), so it seems that the two typing methods yield comparable results for determining genetic relationships between isolates. We also compared the wgMLST analyses with a core SNP typing method but found no significant differences, except for 2 isolates obtained from one person, but there was a long time between the sample collection dates. The person could still carry the same strain that has adapted and changed or perhaps the person encountered a genetically highly related strain and became reinfected.

In this study, we defined a genetic cluster cutoff value of ≥2 isolates that differ by ≤20 alleles to determine whether the isolates were genetically related. We cannot conclude whether two isolates that form a genetic cluster are the same strain if we rely only on genetic distance. Additional information on the isolates in the genetic cluster is needed, including the number and variety of resistance genes, replicon families, virulence determinants, and also epidemiological and clinical data. Until now, only a few Dutch *S. argenteus* genetic clusters have been investigated; thus, possibly the cluster cutoff may vary in the future.

From six persons multiple *S. argenteus* isolates were obtained over a period of time which revealed that each person carried her or his own unique strain. In other studies, it was also found that *S. argenteus* can persist in a person for longer periods of time (18, 19). The wgMLST showed that some isolates that were genetically closely related were obtained from persons living in the same postal area, suggesting transmission. For two such genetic clusters, SarCluster-007 and SarCluster-008, the persons from whom the MRSArg isolates were obtained were living in the same household, making transmission highly likely.

When comparing the Dutch MRSArg isolates with the international MRSArg isolates, only one genetic cluster was identified with an isolate retrieved from the Netherlands and two isolates sequenced in the United States. One Dutch MRSArg isolate was found in genogroup GG1850 next to Australian MRSArg isolates. There was only one Dutch isolate with this sequence type, and since this person was hospitalized in Australia in the past, an international transmission is likely.

The studied MRSArg isolates from the Netherlands resembled MRSArg isolates from other countries, when their resistance genes were compared. Only a few genes encoding resistance to methicillin, trimethoprim, and beta-lactam were identified, indicating that these MRSArg isolates were not highly resistant. MSSArg isolates were not methicillin resistant and lacked the *dfrG* gene, but >50% of the MSSArg isolates carried the aminoglycoside resistance gene *aph(3)-III* (58.8%) and tetracycline resistance gene *tet(L)* (53.7%). When the virulence determinants were compared between MRSArg isolates from the Netherlands and MRSArg isolates from other countries, only small differences were found. Most of the Dutch MRSArg isolates carried the immunomodulating genes *sac* and *scn*, suggesting that the isolates are as virulent as the MRSArg and MSSArg isolates from other countries. The enterotoxins identified in *S. argenteus* are different from those in S. aureus but similar to the enterotoxins reported in *S. argenteus* by Wakabayashi et al. (12). These enterotoxin-encoding genes occur in 25% of the MRSArg isolates but in only 9.6% of the MSSArg isolates. Some studies reported PVL-encoding genes in MSSArg isolates (3, 8, 11, 16, 19) but not in MRSArg isolates (3, 18); since we studied only MRSArg isolates, we did not observe these.

The isolates were identified as *S. argenteus* by mass spectrometry (MALDI-ToF MS), but since the discrimination between the different species belonging to the S. aureus complex is not very reliable, we also used the core genes present in the *S. argenteus* and S. aureus wgMLST scheme to confirm the species identification. While doing so, we noticed that the core genes found in *S. argenteus* differed considerably from those found in S. aureus. Also, the other members of the SAC, *S. schweitzeri*, *S. roterodami*, and *S. singaporensis*, seemed to have different core genes when they were analyzed with the S. aureus and *S. argenteus* wgMLST scheme. This shows that the five species belonging to the S. aureus complex differ significantly in their core genes and that the wgMLST scheme designed for *S. argenteus* is highly specific for *S. argenteus.* This finding puts some doubt on the grouping of these five species in a single complex. For *S. roterodami* only one reference strain was available (EMCR19), so this is a limitation. When more *S. roterodami* sequences are available, it may be interesting to analyze them as well to see if they have the same number of core genes for the two schemes as EMCR19.

In conclusion, MRSArg appears to be a unique pathogen sharing important resistance and virulence characteristics with MRSA. In order to gain insights in the prevalence and clinical relevance of MRSArg in the Netherlands, the Dutch national MRSA surveillance has been expanded to include other methicillin-resistant members of the S. aureus complex, including *S. schweitzeri* and *S. argenteus*.

## MATERIALS AND METHODS

### Bacterial isolates.

The National Institute for Public Health and the Environment (RIVM) performs the national MRSA surveillance in the Netherlands. Dutch medical microbiology laboratories (MMLs) submit MRSA isolates to the RIVM, where they are typed by MLVA (21). Some of the isolates are not Staphylococcus aureus and cannot be typed using MLVA, since they lack more than two of the eight required variable-number tandem repeat loci. mass spectrometry (MALDI-ToF MS) mass spectrometry (MS) microflex LT (Bruker, Billerica, MA, USA) with the December 2018 updated database including the *S. argenteus* spectrum (BDAL version 8.0.0.0 & SR 1.0.0.0 & in-house; Bruker, Billerica, MA, USA) was performed on 64 isolates.

The submitted isolates were accompanied with an epidemiological questionnaire containing patient characteristics, but for some of the older isolates the questionnaire was only partially filled in (Table 1).

### Whole-genome sequencing.

NGS was performed using a NovaSeq 6000 machine (Illumina, San Diego, CA, USA) yielding 150-base-long paired-end reads. The NGS data were used to create contigs, using CLC Genomics Workbench v20.0.3 (Qiagen Bioinformatics, Aarhus, Denmark), and only contigs with a minimum length of 500 bases and a read coverage of >30 were used for analysis. Three isolates, RIVM_M020832, RIVM_M036020, and RIVM_M046968, were also sequenced with Nanopore long-read sequencing (Oxford Nanopore Technologies, Oxford, United Kingdom). Chromosomes and plasmids were reconstructed using Unicycler hybrid assembly, and the resulting contigs were annotated with Prokka as described previously (22) and submitted to NCBI.

### Genomic analyses.

An *S. argenteus*-specific wgMLST scheme was designed in SeqSphere v3.5.0 (Ridom, Münster, Germany) using the annotated chromosome sequence of isolate CP086572 as the seed sequence and the two other isolates CP086570 and CP086574 as query genomes. This yielded a wgMLST scheme comprising 2,168 core genes and 387 accessory genes. All *S. argenteus* isolates were analyzed with this wgMLST scheme, and each isolate yielded more than 95% good targets, which means that 95% of the genes from the core genome were present in each isolate.

Ten MRSA isolates from our collection were analyzed with the in-house *S. argenteus* wgMLST scheme, and this showed only 34.9% of the *S. argenteus* core genes present in S. aureus. Nine available *S. schweitzeri* sequences ware analyzed and yielded 77.0% of the core genes present in the *S. argenteus* wgMLST scheme. And the recently described *S. roterodami* EMCR19, which is only one isolate, and 6 available sequences of the newly described *S. singaporensis* both yielded 87.3% of the core genes present in the *S. argenteus* wgMLST scheme. The same sequences were also analyzed with the wgMLST scheme for S. aureus. Here, we found that the S. aureus isolates scored more than 96.4% of the core genes present in the wgMLST scheme for S. aureus, but *S. schweitzeri* yielded 47.0%, *S. roterodami* yielded 39.3%, and *S. singaporensis* yielded only 37.7% of the core genes present in the S. aureus wgMLST scheme.

Apart from the Dutch isolates, we used the NCBI (129 isolates) and SRA (38 isolates) databases to obtain NGS data from *S. argenteus* isolates studied in other countries. Of 129 NCBI isolates 30 were MRSArg and 99 were methicillin-sensitive *S. argenteus* (MSSArg). Of 38 SRA isolates two were MRSArg and 36 were MSSArg. The in-house *S. argenteus* wgMLST scheme was used to compare international *S. argenteus* isolates with the isolates retrieved in the Netherlands. Next, the allelic profiles were imported in BioNumerics (v7.6.3; Applied Maths, Sint-Martens-Latem, Belgium) and used to assess genetic relationships between isolates, which were visualized in a minimum spanning tree (MST). Missing alleles were ignored and not counted as allelic differences. A genetic cluster is defined as ≥2 isolates that differ by ≤20 alleles, and genogroups differ by ≥1,800 alleles from each other.

All 54 Dutch isolates were typed using an SNP typing tool of CLC Genomics Workbench v20.0.3, using CP086572 as a reference. The core genome was annotated and used for core SNP typing, excluding the variable regions.

ResFinder, PlasmidFinder, VirulenceFinder, and SCCmec Finder databases from the Center for Genomic Epidemiology (https://bitbucket.org/genomicepidemiology, downloaded on 1 April 2020) were used for the identification of resistance genes, replicons, virulence determinants, and the SCC*mec* type, respectively. A threshold of 95% was used for identity and 60% for the minimum length for ResFinder and PlasmidFinder. Targets that had less than 100% identity with the ResFinder, VirulenceFinder, or PlasmidFinder database reference gene for the specific resistance genes, virulence genes, or replicons were further analyzed by mapping Illumina reads to the specific reference gene to determine whether the found differences were silent mutations or caused amino acid changes.

### Ethics statement.

The bacterial isolates belong to the medical microbiological laboratories participating in the Dutch national MRSA surveillance program and were obtained as part of routine clinical care in the past years. Since no identifiable personal data were collected and data were analyzed and processed anonymously, written or verbal patient consent was not required. According to the Dutch Medical Research Involving Human Subjects Act (WMO), this study was exempt from review by an Institutional Review Board.

### Data availability.

The Illumina (NGS) sequence data set generated and analyzed in this study is available in the Sequence Read Archive (SRA) in project PRJNA794761 (accession numbers SRR17445809 through SRR17445863). The plasmid and chromosome sequences are deposited in GenBank of the National Center for Biotechnology Information (NCBI) and available through the accession numbers CP086570, CP086572, and CP086574. We confirm that all supporting data, code, protocols, and accession numbers have been provided within the article and through supplemental material.
