# Supplementary material for: Genetic Characteristics of Methicillin-Resistant Staphylococcus argenteus Isolates Collected in the Dutch National MRSA Surveillance from 2008 to 2021
Source: Microbiol Spectr. 2022 Aug 25;10(5):e01035-22. doi: 10.1128/spectrum.01035-22 (PMC9603934; doi:10.1128/spectrum.01035-22)
Supplement: Supplemental file 1 — Supplemental material. Download spectrum.01035-22-s0001.pdf, PDF file, 0.3 MB [file spectrum.01035-22-s0001.pdf]

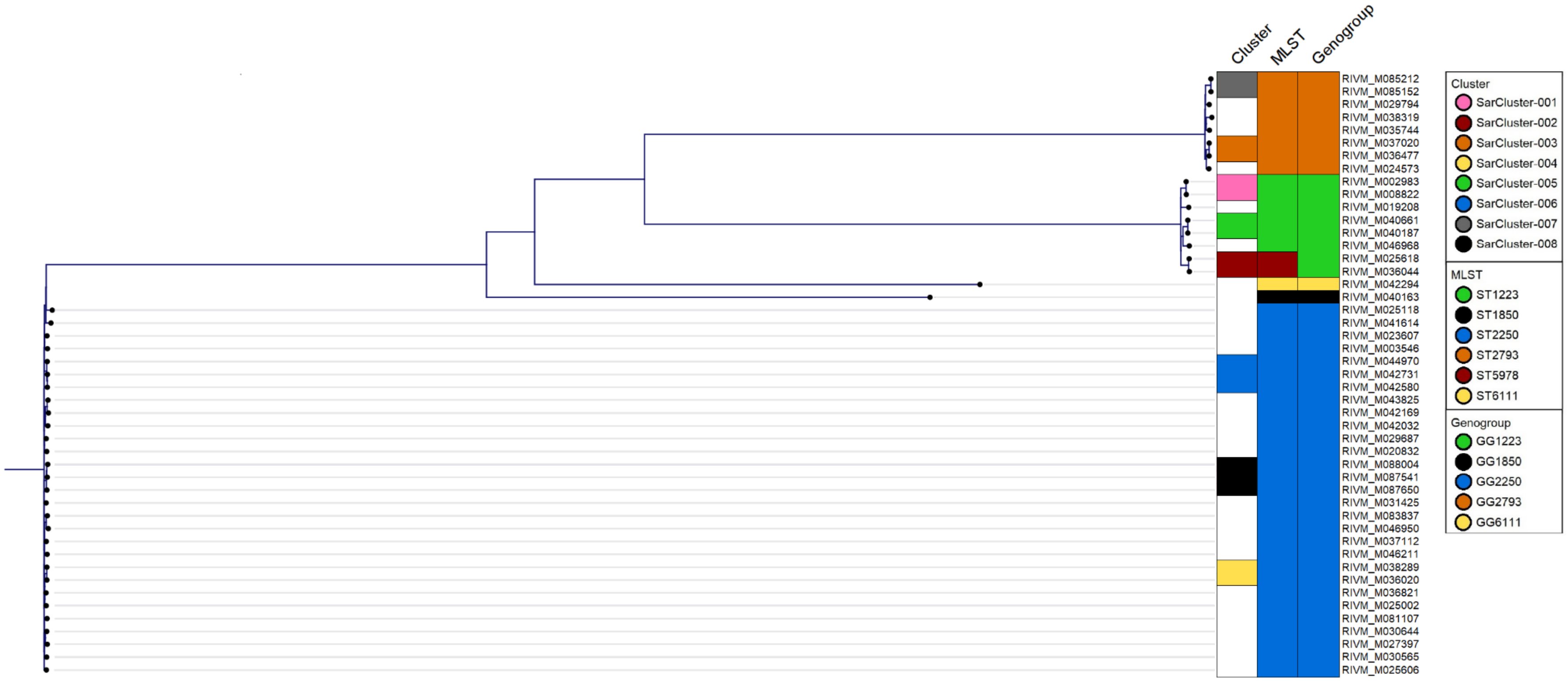

Supplemental material Figure 1. Single nucleotide polymorphism (SNP) analysis on the 47 unique Dutch *S. argenteus* isolates.

Supplemental material Table 1. SNP-derived matrix showing SNP distances between the 54 *S. argenteus* isolates, including the multiple isolates from one person.

Isolate	RIVM_M002983	RIVM_M003546	RIVM_M008822	RIVM_M011864	RIVM_M019208	RIVM_M020832	RIVM_M023607	RIVM_M024573	RIVM_M025002	RIVM_M025118	RIVM_M025606	RIVM_M025618	RIVM_M026823	RIVM_M027397	RIVM_M029687	RIVM_M029794	RIVM_M030565	RIVM_M030644	RIVM_M031425	RIVM_M031426	RIVM_M035744	RIVM_M036020	RIVM_M036044	RIVM_M036477	RIVM_M036821	RIVM_M037020	RIVM_M037112	RIVM_M038289	RIVM_M038319	RIVM_M040163	RIVM_M040187	RIVM_M040661	RIVM_M040662	RIVM_M041166	RIVM_M041614	RIVM_M042032	RIVM_M042169	RIVM_M042294	RIVM_M042580	RIVM_M042731	RIVM_M043825	RIVM_M044970	RIVM_M045471	RIVM_M046211	RIVM_M046343	RIVM_M046950	RIVM_M046968	RIVM_M081107	RIVM_M083837	RIVM_M085152	RIVM_M085212	RIVM_M087541	RIVM_M087650
RIVM_M002983																																																					
RIVM_M003546	14151																																																				
RIVM_M008822	5	14152																																																			
RIVM_M011864	14167	30	14168																																																		
RIVM_M019208	184	14199	185	14191																																																	
RIVM_M020832	14166	81	14167	73	14190																																																
RIVM_M023607	14162	67	14163	59	14186	68																																															
RIVM_M024573	13788	14456	13789	14450	13807	14449	14447																																														
RIVM_M025002	14159	74	14160	66	14183	57	61	14443																																													
RIVM_M025118	14164	123	14165	135	14208	134	130	14469	127																																												
RIVM_M025606	14163	78	14164	70	14187	61	65	14448	52	131																																											
RIVM_M025618	180	14198	181	14190	208	14189	14185	13811	14182	14207	14186																																										
RIVM_M026823	14164	123	14165	135	14208	134	130	14469	127	2	131	14207																																									
RIVM_M027397	14175	90	14176	82	14199	73	77	14458	64	143	68	14198	143																																								
RIVM_M029687	14159	74	14160	66	14183	51	61	14442	52	127	56	14182	127	68																																							
RIVM_M029794	13779	14445	13780	14457	13814	14454	14454	107	14450	14458	14455	13818	14458	14465	14449																																						
RIVM_M030565	14166	83	14167	75	14190	66	70	14451	57	136	61	14189	136	63	61	14458																																					
RIVM_M030644	14170	85	14171	77	14194	68	72	14449	59	138	63	14193	138	43	63	14456	58																																				
RIVM_M031425	14157	73	14158	65	14181	56	60	14441	47	126	51	14180	126	63	51	14448	56	58																																			
RIVM_M031426	14157	73	14158	65	14181	56	60	14441	47	126	51	14180	126	63	51	14448	56	58	2																																		
RIVM_M035744	13783	14446	13784	14458	13818	14455	14455	107	14451	14459	14456	13822	14459	14466	14450	96	14459	14457	14449	14449																																	
RIVM_M036020	14164	79	14165	71	14188	64	66	14448	41	132	59	14187	132	71	55	14455	64	66	54	54	14456																																
RIVM_M036044	183	14201	184	14193	211	14192	14188	13814	14185	14210	14189	19	14210	14201	14185	13821	14192	14196	14183	14183	13825	14190																															
RIVM_M036477	13789	14456	13790	14450	13808	14449	14447	71	14443	14469	14448	13812	14469	14458	14442	106	14451	14449	14441	14441	106	14448	13815																														
RIVM_M036821	14157	72	14158	64	14181	57	59	14441	34	125	52	14180	125	64	50	14448	57	59	47	47	14449	39	14183	14441																													
RIVM_M037020	13790	14457	13791	14451	13809	14450	14448	72	14444	14470	14449	13813	14470	14459	14443	107	14452	14450	14442	14442	107	14449	13816	1	14442																												
RIVM_M037112	14161	78	14162	70	14185	61	65	14445	48	131	56	14184	131	68	56	14452	61	63	51	51	14453	55	14187	14445	48	14446																											
RIVM_M038289	14164	79	14165	71	14188	64	66	14448	41	132	59	14187	132	71	55	14455	64	66	54	54	14456	2	14190	14448	39	14449	55																										
RIVM_M038319	13810	14475	13811	14487	13845	14484	14484	135	14480	14488	14485	13849	14488	14495	14479	124	14488	14486	14478	14478	104	14485	13852	134	14478	135	14482	14485																									
RIVM_M040163	14223	11047	14224	11045	14249	11044	11040	14717	11037	11061	11037	14253	11061	11053	11037	14724	11046	11048	11036	11036	14726	11042	14256	14717	11035	14718	11037	11042	14756																								
RIVM_M040187	164	14178	165	14170	192	14169	14165	13791	14162	14187	14166	184	14187	14178	14162	13798	14169	14173	14160	14160	13802	14167	187	13792	14160	13793	14164	14167	13829	14232																							
RIVM_M040661	164	14178	165	14170	192	14169	14165	13791	14162	14187	14166	184	14187	14178	14162	13798	14169	14173	14160	14160	13802	14167	187	13792	14160	13793	14164	14167	13829	14232	0																						
RIVM_M040662	163	14177	164	14171	193	14170	14166	13790	14163	14186	14167	185	14186	14179	14163	13797	14170	14174	14161	14161	13801	14168	188	13791	14161	13792	14165	14168	13828	14233	5	5																					
RIVM_M041166	14223	11047	14224	11045	14249	11044	11040	14717	11037	11061	11037	14253	11061	11053	11037	14724	11046	11048	11036	11036	14726	11042	14256	14717	11035	14718	11037	11042	14756	0	14232	14232	14233																				
RIVM_M041614	14174	128	14175	120	14198	117	115	14461	112	145	116	14197	145	128	112	14466	121	123	111	111	14467	117	14200	14461	110	14462	116	117	14496	11057	14177	14177	14178	11057																			
RIVM_M042032	14175	92	14176	84	14199	73	79	14460	68	145	72	14198	145	84	68	14467	77	79	67	67	14468	75	14201	14460	68	14461	72	75	14497	11053	14178	14178	14179	11053	130																		
RIVM_M042169	14177	97	14178	89	14201	78	84	14464	73	150	77	14200	150	89	73	14471	82	84	72	72	14472	80	14203	14464	73	14465	77	80	14501	11058	14180	14180	14181	11058	135	63																	
RIVM_M042294	13606	11617	13607	11609	13626	11606	11604	14062	11601	11626	11603	13628	11626	11617	11599	14070	11610	11612	11600	11600	14072	11606	13631	14063	11599	14064	11603	11606	14100	11585	13611	13611	13612	11585	11619	11617	11621																
RIVM_M042580	14167	83	14168	75	14191	74	70	14452	67	136	71	14190	136	83	67	14459	76	78	66	66	14460	72	14193	14452	65	14453	71	72	14489	11045	14170	14170	14171	11045	121	85	90	11609															
RIVM_M042731	14168	84	14169	76	14192	75	71	14451	68	137	72	14191	137	84	68	14458	77	79	67	67	14459	73	14194	14451	66	14452	72	73	14488	11046	14171	14171	14172	11046	122	86	91	11610	3														
RIVM_M043825	14176	95	14177	87	14200	76	82	14463	71	148	75	14199	148	87	71	14470	80	82	70	70	14471	78	14202	14463	71	14464	75	78	14500	11056	14179	14179	14180	11056	133	61	22	11620	88	89													
RIVM_M044970	14166	82	14167	74	14190	73	69	14451	66	135	70	14189	135	82	66	14458	75	77	65	65	14459	71	14192	14451	64	14452	70	71	14488	11044	14169	14169	14170	11044	120	84	89	11608	13	14	87												
RIVM_M045471	14225	11049	14226	11047	14251	11046	11042	14719	11039	11063	11039	14255	11063	11055	11039	14726	11048	11050	11038	11038	14728	11044	14258	14719	11037	14720	11039	11044	14758	2	14234	14234	14235	2	11059	11055	11060	11587	11047	11048	11058	11046											
RIVM_M046211	14173	88	14174	80	14197	73	75	14457	60	141	68	14196	141	80	64	14464	73	75	63	63	14465	61	14199	14457	58	14458	56	61	14494	11051	14176	14176	14177	11051	126	84	89	11613	81	82	87	80	11053										
RIVM_M046343	14172	87	14173	79	14196	72	74	14456	59	140	67	14195	140	79	63	14463	72	74	62	62	14464	60	14198	14456	57	14457	55	60	14493	11050	14175	14175	14176	11050	125	83	88	11612	80	81	86	79	11052	1									
RIVM_M046950	14190	105	14191	97	14214	88	92	14473	75	158	83	14213	158	95	83	14480	88	90	78	78	14481	82	14216	14473	75	14474	71	82	14510	11067	14191	14191	14192	11067	143	99	104	11631	98	99	102	97	11069	83	82								
RIVM_M046968	180	14196	181	14188	208	14187	14183	13811	14180	14205	14184	200	14205	14196	14180	13818	14187	14191	14178	14178	13822	14185	203	13812	14178	13813	14182	14185	13849	14250	138	138	141	14250	14195	14196	14197	13627	14188	14189	14196	14187	14252	14194	14193	14211							
RIVM_M081107	14169	85	14170	77	14193	70	72	14452	61	138	65	14192	138	67	61	14459	60	62	60	60	14460	62	14195	14452	59	14453	65	62	14489	11045	14172	14172	14173	11045	123	81	86	11609	78	79	84	77	11047	71	70	92	14190						
RIVM_M083837	14176	91	14177	83	14200	74	78	14459	61	144	69	14199	144	81	69	14466	74	76	64	64	14467	68	14202	14459	61	14460	57	68	14496	11053	14177	14177	14178	11053	129	85	90	11617	84	85	88	83	11055	69	68	42	14197	78					
RIVM_M085152	13805	14467	13806	14479	13840	14478	14478	140	14474	14482	14479	13846	14482	14489	14473	115	14482	14484	14472	14472	129	14479	13849	139	14472	140	14476	14479	157	14751	13826	13826	13825	14751	14490	14491	14495	14093	14483	14482	14494	14482	14753	14488	14487	14504	13846	14483	14490				
RIVM_M085212	13804	14466	13805	14478	13839	14477	14477	139	14473	14481	14478	13845	14481	14488	14472	114	14481	14483	14471	14471	128	14478	13848	138	14471	139	14475	14478	156	14750	13825	13825	13824	14750	14489	14490	14494	14092	14482	14481	14493	14481	14752	14487	14486	14503	13845	14482	14489	1			
RIVM_M087541	14173	88	14174	80	14197	71	75	14458	62	141	66	14196	141	78	66	14465	71	73	61	61	14466	69	14199	14458	62	14459	66	69	14495	11051	14176	14176	14177	11051	126	82	87	11615	81	82	85	80	11053	78	77	93	14194	75	79	14489	14488		
RIVM_M087650	14170	85	14171	77	14194	68	72	14455	59	138	63	14193	138	75	63	14462	68	70	58	58	14463	66	14196	14455	59	14456	63	66	14492	11048	14173	14173	14174	11048	123	79	84	11612	78	79	82	77	11050	75	74	90	14191	72	76	14486	14485	17	
RIVM_M088004	14177	93	14178	85	14201	76	80	14462	67	146	71	14200	146	83	71	14469	76	78	66	66	14470	74	14203	14462	67	14463	71	74	14499	11055	14180	14180	14181	11055	131	87	92	11619	86	87	90	85	11057	83	82	98	14198	80	84	14493	14492	17	22
